# Negative Life Events and Emotional Symptoms From Ages 2 to 30 Years

**DOI:** 10.1001/jamanetworkopen.2024.29448

**Published:** 2024-08-29

**Authors:** William E. Copeland, Ryan Keen, Guangyu Tong, Lilly Shanahan

**Affiliations:** 1 Department of Psychiatry, University of Vermont, Burlington; 2 Harvard University, Cambridge, Massachusetts; 3 Department of Internal Medicine and Biostatistics, Yale University, New Haven, Connecticut; 4 Department of Psychology, University of Zurich, Zürich, Switzerland

## Abstract

**Importance:**

Exposure to different types of negative life events, including traumatic events, is common across the lifespan and associated with increased mental health symptoms.

**Objective:**

To assess whether vulnerability to negative life events varies across 5 developmental periods from preschool to young adulthood.

**Design, Setting, and Participants:**

This cohort study analyzed data from 3 community-representative studies set in the southeastern US (1992-2015) with harmonized assessment approaches that included a total of 13 775 assessments of individuals aged 2 to 30 years with up to 21 years of follow-up. Data analysis occurred from July 2023 to June 2024.

**Exposures:**

Each study assessed lifetime exposure to (1) traumatic events (ie, severe events associated with posttraumatic stress disorder) and (2) recent stressful events (eg, loss of a friend or moving). All assessments were completed with structured interviews with participants and/or their caregivers.

**Main Outcomes and Measures:**

The primary outcome was emotional symptoms (ie, anxiety and depressive symptoms). Associations of both categories of life events with emotional symptoms were compared across preschool (<7 years), childhood (7-12 years), adolescence (13-17 years), late adolescence (18-22 years), and young adulthood (23-30 years).

**Results:**

Analyses were based on 13 775 assessments of 3258 participants (1519 female [weighted percentage, 50.0%]). Recent stressful events were associated with emotional symptoms across each developmental period, ranging from a low in preschool (*B* =0.14; SE = 0.05) to a high in young adulthood (*B* = 0.57; SE = 0.12) in cross-sectional analyses and ranging from a low in childhood (*B* = 0.10; SE = 0.06) to a high adolescence (*B* = 0.19; SE = 0.05) in longitudinal analyses. Lifetime traumatic events were associated with emotional symptoms across each developmental period, ranging from a low in preschool (*B* = 0.18; SE = 0.05) to a high in adolescence (*B* = 0.28; SE = 0.04) in cross-sectional analyses and ranging from a low in childhood (*B* = 0.09; SE = 0.06) to a high in late adolescence (*B* = 0.21; SE = 0.05) in longitudinal analyses. Associations had overlapping 95% CIs across the different developmental periods with one exception: stressful events had a larger-magnitude cross-sectional association with emotional symptoms in young adulthood than in other developmental periods. Results were consistent with additive, rather than interactive, associations of traumatic and stressful events with emotional symptoms at each developmental period.

**Conclusions and Relevance:**

In this cohort study of 3 community-representative samples, vulnerability to traumatic and stressful events was generally similar across the first 3 decades of life; both types of events had an independent association with emotional functioning. These findings suggest response to stressful events is similar from childhood to adulthood.

## Key Points

**Question:**

Does vulnerability to negative life events vary across 5 developmental periods from preschool to adulthood?

**Findings:**

In this cohort study of 3258 participants from 3 community-representative samples with harmonized assessments, associations of negative life events with emotional symptoms were consistent and typically small to moderate in size across the 5 developmental periods. An exception was young adulthood (ages 23 to 30 years), where these associations were somewhat larger in magnitude than at most other ages.

**Meaning:**

These findings suggest that vulnerability to negative life events is consistent across the lifespan.

## Teaser

This cohort study assesses whether associations of negative life events with emotional symptoms vary across different stages of development from preschool to young adulthood.

## Introduction

The impact of negative life events on mental health is a heavily studied topic in developmental science. Life events broadly include stressful events,^1,2^ traumatic events,^3,4^ and adverse childhood experiences.^5,6^ Research has also identified dimensions of such events that may be particularly deleterious, including loss,^7,8^ threat or danger,^9,10^ deprivation,^11^ and unpredictability.^12^ Extensive research shows that negative life events generally first occur early in life, increase risk for emotional symptoms, and potentially impact long-term functioning. However, it remains unclear whether the developmental period (eg, preschool, childhood, or adulthood) in which an individual is exposed to a negative life event influences the association of such events with emotional symptoms.

Existing evidence has shown that lower-impact life events (ie, stressful events), such as parent separation, are common experiences across the lifespan. Recent evidence also indicates that higher-impact life events (ie, traumatic events) have become more common across childhood and adolescence.^3,13^ Given the widespread and lifelong exposure to negative life events, it is paramount to understand when individuals are most vulnerable to their harmful impact, regardless of when they were first exposed.

Prospective-longitudinal studies examining whether associations of negative life events with emotional symptoms vary by developmental period are rare and often subject to several limitations. For example, one study^14^ applied a structured life course modeling approach to data from the Avon Longitudinal Study of Parents and Children (7476 participants) and found that recent and cumulative exposures to adverse events had the largest magnitude associations with child psychopathology symptoms. However, because all measures were assessed by 8 years of age, the study^14^ only evaluated vulnerability to negative life events during childhood. Relatedly, studies assessing the role of developmental timing across the lifespan have largely relied on retrospective reports assessed in adulthood. Such long-term retrospective recall may be subject to recall bias and forgetting.^15,16,17,18^ Furthermore, telescoping—perceiving distant events as more recent—may prompt misclassification of the timing of exposure.^19,20^ Prospective studies and studies with short time windows between exposure and outcome assessments are well-positioned to limit these biases.

To test variability in vulnerability by age-defined developmental periods, the current analysis uses 3 community-representative studies,^21,22,23^ each of which assessed a range of negative life events and emotional symptoms using harmonized diagnostic interviews. We first hypothesized modestly increased vulnerability to negative life events earlier in life because children have not fully developed the key cognitive, language, and emotional regulation skills that aid in coping. Second, because adolescence is a transitional period marked by substantial development of the reward and emotion regulation systems and by the physiological and social changes that accompany puberty, we also hypothesized increased vulnerability to negative life events during adolescence.^24,25^ Finally, we tested vulnerability models separately for male and female adolescents—the period when sex differences in emotional symptoms emerge.^26,27^

## Methods

This cohort study was approved by/determined exempt from review by the Duke University institutional review board and followed the Strengthening the Reporting of Observational Studies in Epidemiology (STROBE) reporting guideline. This study used data from 3 community-based studies^21,22,23^ of mental health with harmonized diagnostic interviews across 5 age-defined developmental periods: (1) preschool (<7 years), (2) childhood (≥7 to <13 years), (3) adolescence (≥13 to <18 years), (4) late adolescence (≥18 to <23 years), and (5) young adulthood (≥23 to 30 years).

The Duke Preschool Anxiety Study (DPAS)^21^ is a cross-sectional study of a representative sample of preschoolers (aged 2 to 5 years) attending a large primary care pediatric clinic in central North Carolina between 2007 and 2010. Screenings of emotional problems were completed for 3424 attendees. Parents of all attendees scoring 4 or more on the screener plus a 7.3% random sample of the remaining attendees were recruited. The assessment included the structured diagnostic interview, the Preschool Age Psychiatric Assessment (PAPA), which assessed emotional symptoms and negative life events. This sample provides observations during the preschool period.

The Great Smoky Mountains Study^22^ (GSMS) is a prospective, population-based study of children in 11 predominantly rural counties in western North Carolina that began in 1993. Three cohorts of children—ages 9, 11, and 13 years—were recruited from a pool of some 20 000 children using a 2-stage sampling design. Annual assessments were completed until 16 years of age. Assessments then continued at ages 19, 21, 26, and 30 years. Assessments included the structured diagnostic interview, the Child and Adolescent Psychiatric Assessment (CAPA) and the Young Adult Psychiatric Assessment (YAPA), which assessed emotional symptoms and negative life events. This sample provides observations during childhood, adolescence, late adolescence, and young adulthood.

The Caring for Children in the Community (CCC) study^23^ is a prospective, population-based study of children aged 9 to 17 years from 4 rural counties in North Carolina between 1997 and 2000. A random sample of 17 117 children and adolescents in the public schools database generated a screening sample of 4500 youths. In addition to initial assessment, 2 additional assessments were completed at 9-month intervals for participants who were younger than 18 years. Assessments included the CAPA structured diagnostic interview, which assessed emotional symptoms and negative life events. This sample provides observations during childhood, adolescence, and late adolescence.

### Measures

Diagnostic interviews for all participants across all 3 studies were based on the structured CAPA for school-age children,^28,29^ its downward extension for preschool children (PAPA),^30^ and its upward extension for young adults (YAPA).^31^ Prior to all interviews, the parent and/or child signed informed consent or assent forms approved by the Duke University Medical Center institutional review board. All interviewers had at least bachelor’s level degrees and received 1 month of training and regular quality control.

For all interviews, the time frame for determining the presence of psychiatric symptoms was the preceding 3 months. For school-age assessments with 2 informants (ie, CAPA in GSMS and CCC), a symptom was marked present if the parent and/or child endorsed it. Scoring programs (written in SAS version 9.4 [SAS Institute]) combined information about the onset, duration, and intensity of each symptom to create *Diagnostic and Statistical Manual of Mental Disorders* (Fourth Edition; *DSM-IV*) diagnoses. Test-retest reliability and validity of the CAPA diagnoses are similar to other established psychiatric interviews.^28,29^ For our analyses, we focused on nonoverlapping symptoms of emotional disorders which include all anxiety (eg, separation anxiety, generalized anxiety, social phobia, or panic) and mood disorders (eg, major depressive disorder and dysthymia). This scale allowed for a similar set of symptoms that are closely associated with negative life events to be compared from preschool to young adulthood.

Exposures to negative events were assessed using the life events modules of the PAPA, CAPA, and YAPA.^32^ For this study, we examined 2 sets of events: (1) lifetime traumatic events defined by *DSM-IV* as precursors of posttraumatic stress disorder (eg, physical abuse, rape, or natural disaster) and (2) recent stressful events covered by most life-event scales to research depression and anxiety (eg, moving, parental divorce, or serious reduction in standard of living). Events are presented in eTable 1 in Supplement 1; events specific to 1 developmental period are noted. For traumatic events, we focused on the number of discrete events reported to date in one’s lifetime because such events are considered sufficiently severe to impact long-term functioning. For stressful events, we focused on the number of discrete events in the past 3 months or recent events because their comparatively lower severity implies that long-term effects are rarely observed.

### Statistical Analysis

The primary analytic approach was a model with current emotional symptoms regressed on the life events variable; this model included adjustments for sex, age at the respective assessment, and race and ethnicity. Race and ethnicity categories across the three studies included African American, American Indian, White, and other (defined as Asian and Hispanic). Race and ethnicity were included in this analysis to insure diversity of the sample and to account for disparities. Emotional symptoms best fit a negative binomial distribution model with a log link. Models were fit in SAS version 15.1 using generalized estimating equations with exchangeable correlation structure and robust variance (sandwich type) estimates to account for within-group correlations that may arise from repeated observations on the same participant.^33^ Sampling weights were incorporated to account for the 2-stage sample designs in all studies.^33^

To test for differences between developmental periods, observations across studies were merged into a single dataset and 3 variables were added to the aforementioned model: (1) study (ie, DPAS vs GSMS vs CCC), (2) developmental period, and (3) an interaction term between the life event and developmental period variables. This interaction term enabled us to test whether the associations of negative life events with emotional symptoms varied by developmental periods. With 5 developmental periods tested, this modeling strategy yielded 10 paired comparisons for each type of life event (ie, traumatic vs stressful event), or 20 comparisons in total. All analyses were tested first in cross-sectional models in which negative life events and emotional symptoms were assessed at the same time point. Analyses were then tested in longitudinal models in which negative events were assessed at 1 time point prior to emotional symptoms (eg, negative life events at time 1 and emotional symptoms at time 2). To preserve the type I error of the global null hypothesis across all tested associations, a Bonferroni correction was applied, resulting in an adjusted 2-sided α of .0025 (calculated as .05/20).^34^ Analyses occurred between July 2023 and June 2024.

## Results

Analyses were based on 13 775 assessments of 3258 participants (1519 female [weighted percentage, 50.0%]). Table 1 compares key features of the 3 included studies. In the DPAS study,^21^ of the 1125 participants recruited, parents of 918 children (81.6%; mean [SD] age, 3.9 [1.3] years; 451 female [weighted percentage, 51.8%]; 388 African American [weighted percentage, 37.5%]; 395 White [weighted percentage, 62.1%]) completed assessments. In the GSMS study,^22^ of the 1777 selected participants, 1420 (79.9%; mean [SD] age, 17.5 [6.2] years; 630 female [weighted percentage, 49.2%]; 88 African American [weighted percentage 6.4%]; 349 American Indian [weighted percentage, 3.8%]; 983 White [weighted percentage 89.8%]) completed interviews. In the CCC study,^23^ of the 1302 individuals selected to participate, 920 (70.7%; mean [SD] age, 14.2 [3.4 years]; 438 female [weighted percentage, 50.0%]; 541 African American [weighted percentage, 53.8%]; 335 White [weighted percentage 41.0%]; 44 other [weighted percentage, 5.2%]) completed assessments. Table 2 provides means and SDs for emotional symptoms, recent (3-month) stressful events, and lifetime traumatic events variables for each study by developmental period combination.

**Table 1.** Comparison of 3 Community-Based Samples

Characteristic	Participants, No. (weighted %)		
	Duke Preschool Anxiety Study (N =918)	Great Smoky Mountains Study (N =1420)	Caring for Children in the Community Study (N =920)
Observations, No.	918	11 230	1627
Informant	Parent report	Parent and self-report	Parent and self-report
Age, mean (SD) [range], y	3.9 (1.3) [2.0-6.0]	17.5 (6.2) [9.0-30.0]	14.2 (3.4) [9.0-17.0]
Sex			
Female	451 (51.8)	630 (49.2)	438 (50.0)
Male	467 (48.2)	790 (50.8)	438 (50.0)
Race and ethnicity^a^			
African American	388 (37.5)	88 (6.4)	541 (53.8)
American Indian	NA	349 (3.8)	NA
White	395 (62.1)	983 (89.8)	335 (41.0)
Other^b^	1 (0.5)	NA	44 (5.2)
Interview	Preschool Age Psychiatric Assessment	Child and Adolescent Psychiatric Assessment and Young Adult Psychiatric Assessment	Child and Adolescent Psychiatric Assessment

Abbreviation: NA, not applicable.

^a^ Race and ethnicity for participants was reported by parents for all studies.

^b^ Other includes Asian and Hispanic.

**Table 2.** Descriptive Statistics for Negative Event and Emotional Symptoms Variables

Developmental period and assessment^a^^,^^b^	Score, mean (SD)		
	Emotional symptoms	3-mo stressful events	Lifetime traumatic events
Preschool: DPAS (n =918)	5.82 (4.04)	1.84 (0.99)	0.63 (0.92)
Childhood			
GSMS (n = 2691)	2.13 (2.71)	0.47 (0.80)	0.55 (0.86)
CCC (n = 605)	4.07 (4.18)	0.52 (0.80)	1.43 (0.90)
Adolescence			
GSMS (n = 3983)	1.78 (2.72)	0.47 (0.80)	0.43 (0.76)
CCC (n = 852)	3.45 (3.92)	0.57 (0.90)	1.59 (1.07)
Late adolescence			
GSMS (n = 2132)	1.70 (3.02)	0.44 (0.66)	0.75 (1.09)
CCC (n = 170)	3.61 (3.75)	0.50 (0.72)	1.85 (1.39)
Young adulthood: GSMS (n = 2424)	2.15 (3.36)	0.18 (0.48)	0.85 (1.10)

Abbreviations: CCC, Caring for Children in the Community Study; DPAS, Duke Preschool Anxiety Study; GSMS, Great Smoky Mountains Study.

^a^ Sample sizes refer to observations and may involve multiple observations of the same individual.

^b^ Preschool includes participants younger than 7 years; childhood, 7 to younger than 13 years; adolescence, 13 to younger than 18 years; late adolescence, 18 to younger than 23 years; and young adulthood, 23 to 30 years.

### Cross-Sectional Analyses

The Figure and Table 3 show the adjusted cross-sectional associations of the stressful events with emotional symptoms in each developmental period. For nearly all stressful events models (Figure, A), associations with emotional symptoms were statistically significant, small in magnitude (negative binomial regression coefficients <0.4) and had overlapping 95% CIs. Recent stressful events were associated with emotional symptoms across each developmental period, ranging from a low in preschool (B =0.14; SE = 0.05) to a high young adulthood (*B* = 0.57; SE = 0.12); however, the association of stressful events with emotional symptoms during young adulthood in the GSMS sample was of moderate magnitude. For traumatic events (Figure, B), all associations were statistically significant (ranging from a low in preschool [*B* = 0.18; SE = 0.05] to a high in adolescence [*B* = 0.28; SE = 0.04]), small in magnitude (negative binomial regression coefficients <0.3) and had overlapping 95% CIs. Results for the pairwise comparisons based on the interaction term (Table 3) provided little evidence that associations varied by developmental group. Notably, however, stressful events had a larger-magnitude association with emotional symptoms in young adulthood (age 23 to 30 years) than in other developmental periods. In follow-up models testing both recent and lifetime traumatic events simultaneously, the regression coefficient associated with recent stressful events (*B* = 0.23; SE = 0.33) was about twice that of lifetime traumatic events (*B* = 0.36; SE = 0.03).

**Figure.**
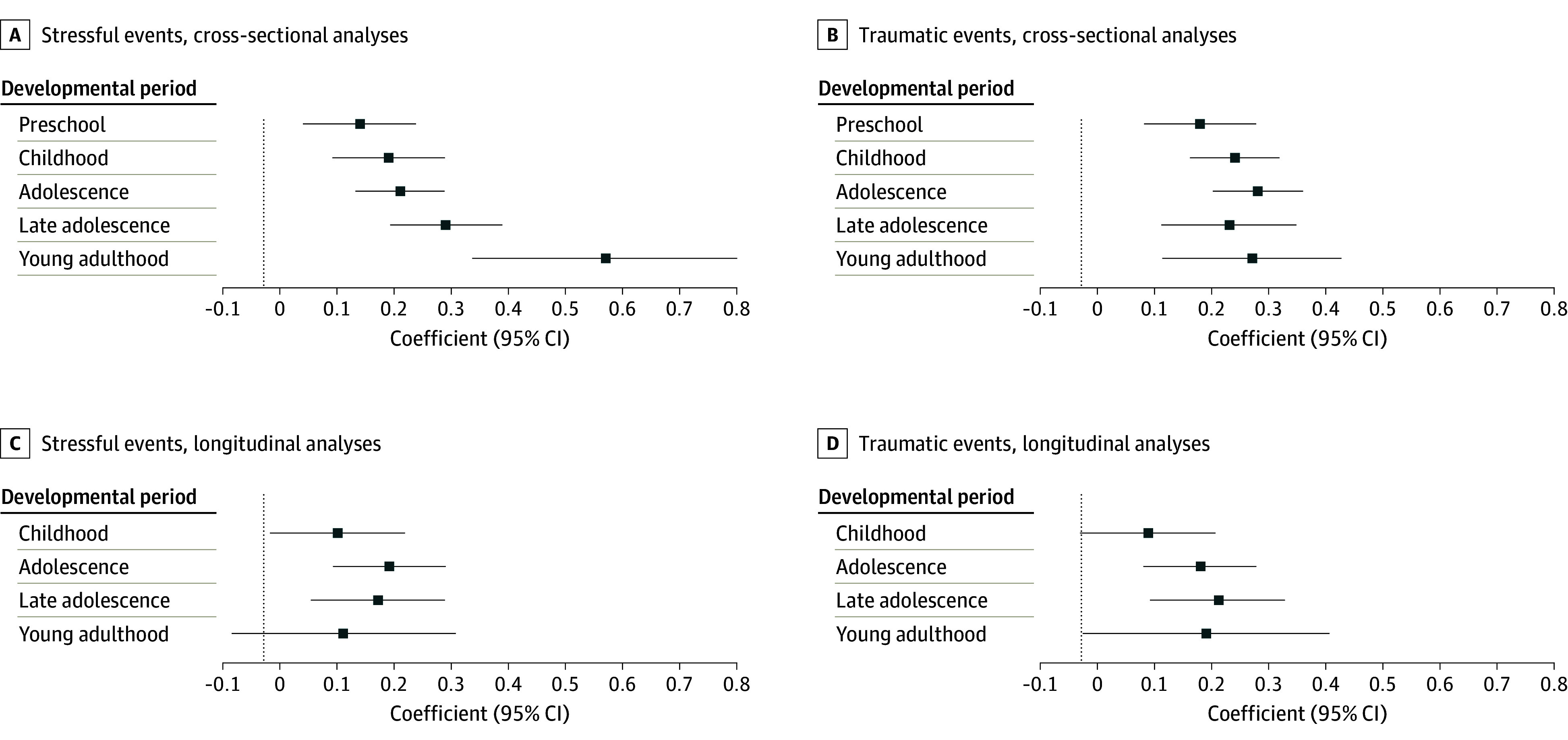
Associations of Number of Negative Life Events With Emotional Symptoms in Different Developmental Periods For developmental groups, preschool includes participants younger than 7 years; childhood includes those aged 7 to younger than 13 years; adolescence includes those aged 13 to younger than 18 years; late adolescence includes those aged 18 to younger than 23 years; and young adulthood includes those aged 23 to 30 years. Results include negative binomial regression coefficients with 95% CIs. Longitudinal analyses only include data from Caring for Children in the Community Study and Great Smoky Mountains Study samples.

**Table 3.** Comparison of Cross-Sectional Event-Symptoms Associations Between Developmental Period Groups

Outcome by developmental period^a^	Regression coefficient		Test of differences by age group, *P* value			
	*B*	SE	Preschool	Childhood	Adolescence	Late adolescence
Stressful events						
Preschool	0.14	0.05	NA	NA	NA	NA
Childhood	0.19	0.05	.79	NA	NA	NA
Adolescence	0.21	0.04	.56	.76	NA	NA
Late adolescence	0.29	0.05	.14	.20	.26	NA
Young adulthood	0.57	0.12	.<.001^b^	<.001^b^	<.001^b^	.02
Overall	0.21	0.03	NA	NA	NA	NA
Traumatic events						
Preschool	0.18	0.05	NA	NA	NA	NA
Childhood	0.24	0.04	.78	NA	NA	NA
Adolescence	0.28	0.04	.39	.39	NA	NA
Late adolescence	0.23	0.06	.70	.86	.52	NA
Young adulthood	0.27	0.08	.83	.99	.61	.90
Overall	0.21	0.03	NA	NA	NA	NA

Abbreviation: NA, not applicable.

^a^ Preschool includes participants younger than 7 years; childhood, 7 to younger than 13 years; adolescence, 13 to younger than 18 years; late adolescence, 18 to younger than 23 years; and young adulthood, 23 to 30 years.

^b^ Meets Bonferroni-corrected α.

### Longitudinal Analyses

Results from the models using only longitudinal observations from the GSMS and CCC (Figure, C and D, and Table 4) were similar to those for cross-sectional analyses with little evidence of differences between developmental groups. Recent stressful events were associated with emotional symptoms across each developmental period, ranging from a low in childhood (*B* = 0.10; SE = 0.06) to a high in adolescence (*B* = 0.19; SE = 0.05). Lifetime traumatic events were also associated with emotional symptoms across each developmental period, ranging from a low in childhood (*B* = 0.09; SE = 0.06) to a high in late adolescence (*B* = 0.21; SE = 0.05). The estimated coefficients were generally more modest in size (≤0.21) than those observed in the cross-sectional analyses.

**Table 4.** Comparison of Longitudinal Event-Symptoms Associations Between Developmental Period Groups

Outcome by developmental period^a^	Regression coefficient		Test of differences by age group, *P* value		
	*B*	SE	Childhood	Adolescence	Late adolescence
Stressful events					
Childhood	0.10	0.06	NA	NA	NA
Adolescence	0.19	0.05	.66	NA	NA
Late adolescence	0.17	0.06	.68	.92	NA
Young adulthood	0.11	0.10	.81	.58	.58
Overall	0.16	0.02	NA	NA	NA
Traumatic events			NA	NA	NA
Childhood	0.09	0.06	NA	NA	NA
Adolescence	0.18	0.05	.18	NA	NA
Late adolescence	0.21	0.06	.16	.47	NA
Young adulthood	0.19	0.11	.34	.37	.97
Overall	0.17	0.02	NA	NA	NA

Abbreviation: NA, not applicable.

^a^ Childhood includes participants aged 7 to younger than 13 years; adolescence, 13 to younger than 18 years; late adolescence, 18 to younger than 23 years; and young adulthood, 23 to 30 years.

### Sensitivity Analyses

Sensitivity analyses tested the robustness of these results to alternative modeling specifications. First, we tested a series of models to examine whether results were similar across depression and anxiety symptoms scales—the scales that were combined for the overall emotional symptoms scale in the main analyses. Results with these scales were similar to those obtained using the overall emotional symptoms variables (eTable 2 and eTable 3 in Supplement 1). Second, while associations of life events with emotional symptoms may be similar across developmental periods, associations of such events with functioning may vary across developmental period due to improvements in coping skills. Therefore, we tested associations of life events with symptom-associated impairment in important life domains (eg, school, work, and peer and family relations). While this impairment variable only had a modest correlation with the total emotional symptom score (*r* = 0.33), results (eTable 4 in Supplement 1) were similar to those observed with symptom variables. Third, because developmental variability (eg, risk for stressful events in adolescence) may be associated with sex, we assessed males and females separately. The results (eTable 5 and eTable 6 in Supplement 1) again remained largely unchanged. Overall, this series of sensitivity analyses suggest that our findings are robust.

### Additive and Interactive Effects

We further tested the effect of both traumatic and stressful events at the same time, as well as an interaction term between these 2 types of events at each time point (eTable 7 in Supplement 1). This model tested (1) whether the associations of traumatic and stressful events were distinct or overlapping and (2) whether there was any evidence of either the stress sensitization or stress inoculation hypotheses. In each developmental period except preschool, both traumatic and stressful events had significant associations with emotional symptoms, suggesting they have additive effects. There was no evidence of an interaction between traumatic and stressful events during any developmental period.

## Discussion

This cohort study combined observations from 3 community-representative samples that collectively included individuals aged 2 to 30 years to test whether the associations of negative life events with emotional symptoms vary by developmental period. There was little evidence that vulnerability to negative life events (ie, traumatic vs stressful) changed between the preschool and young adult ages. The sole exception was a larger-magnitude association of stressful events with emotional symptoms in young adulthood (ages 23 to 30 years). Broadly, these results suggest similar levels of vulnerability to different types of negative life events across the first 3 decades of life.

Numerous related studies have posited differential vulnerability to negative life events during childhood and adolescence.^35,36^ While the empirical evidence is limited, the reasoning is logical. Development involves the acquisition of many socioemotional skills—such as language, fluid intelligence, emotion regulation, and executive and social functioning—that help individuals cope with negative life events.^37^ With these skills remaining underdeveloped prior to adulthood, it is logical that early life developmental periods would be marked by greater vulnerability to negative life events. By extension, adults—given their age—likely have experienced more negative events and thus have better honed their coping skills over time. Such extensive practice may reduce vulnerability to negative life events. Despite this logic, however, we found no evidence that adults are any less susceptible to the harmful effects of negative life events than preschoolers whose skills are still nascent. In fact, young adulthood was the only period that suggested increased vulnerability in this study. These empirical findings directly contradict a key assumption in developmental science: that development matters.

Increased age may present opportunities to improve coping skills; it may also increase one’s cumulative exposure to negative events.^14,38,39^ Multiple exposures may impart wear and tear on the individual that counteracts the benefits of improved coping skills and, consequently, produces a relatively consistent vulnerability over the lifespan. This explanation, however, relies upon parallel, contrasting processes to explain similar vulnerability across development. A more parsimonious explanation is that one’s ability to cope with negative life events may be stable across development. For example, if one’s hand is pinched while closing a door, pain is sure to follow, regardless of age. Likewise, event-induced emotional distress may also be conserved because it too provides information that helps guide individual behaviors. In this sense, it is expected that negative events are associated with increased symptoms at all developmental periods.

While the associations with developmental outcomes were modest, differences between traumatic and stressful events were notable. In both the cross-sectional and the longitudinal models, the associations of stressful events with emotional symptoms increased in young adulthood while the association of traumatic events with symptoms either diminished or remained unchanged. The recency effect may explain such differences. Specifically, it is plausible that emotional functioning may be more susceptible to proximal events (ie, stressful events that occurred within the preceding 3 months) rather than distal events (ie, traumatic events accumulated over the lifespan). Studies have shown that while some events can exert an impact many years after their occurrence, the comparative impact of recent events—even events that are not considered traumatic—is greater.

### Strengths and Limitations

Our study benefits from combining multiple large population-based samples, from obtaining rigorous assessments of emotional symptoms using harmonized diagnostic interviews, and from evaluating 2 distinct types of life events. Nevertheless, several limitations should be considered. First, although all samples were representative of the communities from which the participants were selected, none are representative of the broader US population. Second, because the negative life events measure relied on self and/or parent reports, the recall and accuracy of the exposures cannot be verified. Third, our analysis may be susceptible to unmeasured confounding, such as genetic vulnerability. Fourth, while events are presumed to be negative based upon previous evidence, some may improve emotional symptoms or have no effects at all. Fifth, these samples included cohorts born between 1979 and 2000 and it is possible that vulnerability to negative events may differ across cohorts.

## Conclusions

Exposure to negative life events is common across the first 3 decades of life. Emotional distress in response to such experiences may be common as well. The findings of this cohort study suggest that individuals are vulnerable to negative events throughout the lifespan and to a consistent degree. It does not, however, suggest that all individuals respond to such events in the same way across the lifespan. Our findings provide little evidence that individuals become less vulnerable to negative life events across lifespan development of their own accord. Therefore, research to identify skills that promote resilience may aid interventions to reduce or prevent these harmful outcomes, particularly when it is not possible to change the environment that begets potentially stressful or traumatic experiences.

## Notes

Supplement 1. **eTable 1.** List of Stressful and Traumatic Events **eTable 2.** Comparison of Event-Depressive Symptoms Associations Between Developmental Period Groups **eTable 3.** Comparison of Event-Anxiety Symptoms Associations Between Developmental Period Groups **eTable 4.** Comparison of Event-Impairment Associations Between Developmental Period Groups **eTable 5.** Comparison of Event-Symptoms Associations Between Developmental Period Groups For Females (Includes All Samples) **eTable 6.** Comparison of Event-Symptoms Associations Between Developmental Groups for Males **eTable 7.** Follow-Up Model Testing Additive and Interactive Effects of Traumatic and Stressful Events

Supplement 2.
Data Sharing Statement
