# Supplementary material for: Negative Life Events and Emotional Symptoms From Ages 2 to 30 Years
Source: JAMA Netw Open. 2024 Aug 29;7(8):e2429448. doi: 10.1001/jamanetworkopen.2024.29448 (PMC11362870; doi:10.1001/jamanetworkopen.2024.29448)
Supplement: Supplement 1. — eTable 1. List of Stressful and Traumatic Events eTable 2. Comparison of Event-Depressive Symptoms Associations Between Developmental Period Groups eTable 3. Comparison of Event-Anxiety Symptoms Associations Between Developmental Period Groups eTable 4. Comparison of Event-Impairment Associations Between Developmental Period Groups eTable 5. Comparison of Event-Symptoms Associations Between Developmental Period Groups For Females (Includes All Samples) eTable 6. Comparison of Event-Symptoms Associations Between Developmental Groups for Males eTable 7. Follow-Up Model Testing Additive and Interactive Effects of Traumatic and Stressful Events [file jamanetwopen-e2429448-s001.pdf]

Supplemental Online Content

Copeland WE, Keen R, Tong G, Shanahan L. Negative life events and emotional symptoms from ages 2 to 30 years. *JAMA Netw. Open*. 2024;7(8):e2429448. doi:10.1001/jamanetworkopen.2024.29448

eTable 1. List of Stressful and Traumatic Events
eTable 2. Comparison of Event-Depressive Symptoms Associations Between Developmental Period Groups
eTable 3. Comparison of Event-Anxiety Symptoms Associations Between Developmental Period Groups
eTable 4. Comparison of Event-Impairment Associations Between Developmental Period Groups
eTable 5. Comparison of Event-Symptoms Associations Between Developmental Period Groups For Females (Includes All Samples)
eTable 6. Comparison of Event-Symptoms Associations Between Developmental Groups for Males
eTable 7. Follow-Up Model Testing Additive and Interactive Effects of Traumatic and Stressful Events

This supplemental material has been provided by the authors to give readers additional information about their work.

© 2024 Copeland WE et al. *JAMA Network Open.*

**eTable 1.** List of Stressful and Traumatic Events	
**Lifetime Traumatic Events**	**3-month Stressful Events**
Death of a close relative or friend	New child in home
Witness to a traumatic event	Pregnancy
Diagnosis with a life-threatening illness	Parental separation
Serious accident	Parental divorce
Fire	New parental figure
Exposure to life-threatening toxic agent	Moving
Learning of a traumatic event	Change of school/work other than promotion
War or terrorism	Loss of best friend through move
Cause of death or serious harm	Break-up with best friend
Victim of physical violence	Break-up with boy/girlfriend/spouse
Victim of physical abuse	Parental arrest
Kidnapping or captivity	Reduction in standard of living
Sexual abuse	Forced separation from home
Rape	Death of pet*

* Death of pet was only included in stressful events for preschoolers.

© 2024 Copeland WE et al. *JAMA Network Open.*

eTable 2. Comparison of Event-Depressive Symptoms Associations Between Developmental Period Groups

	Preschool	Childhood	Adolescence	Late adolescence
**Stressful Events**	*p*	*p*	*p*	*p*
Preschool	--			
Childhood	0.36	--		
Adolescence	0.51	0.61	--	
Late adolescence	0.81	0.18	0.23	--
Young Adulthood	0.02	0.0002*	0.0004*	0.02
**Traumatic Events**				
Preschool	--			
Childhood	0.39	--		
Adolescence	0.37	0.91	--	
Late adolescence	0.50	0.74	0.67	--
Young Adulthood	0.49	0.96	0.91	0.88

* Significance reached with Bonferroni-corrected alpha. *Preschool* includes participants < 7 years old, *childhood* includes those ≥ 7 to < 13 years old, *adolescence* includes those ages ≥ 13 to < 18 years old , *late adolescence* includes those ages ≥ 18 to < 23 years old, and *young adulthood* includes those ≥ 23 to 30 years old.

© 2024 Copeland WE et al. *JAMA Network Open.*

**eTable 3.** Comparison of Event-Anxiety Symptoms Associations Between Developmental Period Groups				
	Preschool	Childhood	Adolescence	Late adolescence
**Stressful Events**	*p*	*p*	*p*	*p*
Preschool	--			
Childhood	0.33	--		
Adolescence	0.22	0.83	--	
Late adolescence	0.07	0.30	042	--
Young Adulthood	0.0001*	0.001*	0.003	0.04
**Traumatic Events**				
Preschool	--			
Childhood	0.50	--		
Adolescence	0.07	0.14	--	
Late adolescence	0.63	0.85	0.13	--
Young Adulthood	0.71	0.89	0.32	0.99

* Significance reached with Bonferroni-corrected alpha. *Preschool* includes participants < 7 years old, *childhood* includes those ≥ 7 to < 13 years old, *adolescence* includes those ages ≥ 13 to < 18 years old , *late adolescence* includes those ages ≥ 18 to < 23 years old, and *young adulthood* includes those ≥ 23 to 30 years old.

© 2024 Copeland WE et al. *JAMA Network Open.*

**eTable 4.** Comparison of Event-Impairment Associations Between Developmental Period Groups				
	Preschool	Childhood	Adolescence	Late adolescence
**Stressful Events**	*p*	*p*	*p*	*p*
Preschool	--			
Childhood	0.02	--		
Adolescence	0.009	0.82	--	
Late adolescence	0.07	0.79	0.66	--
Young Adulthood	0.03	0.21	0.22	0.17
**Traumatic Events**				
Preschool	--			
Childhood	0.36	--		
Adolescence	0.22	0.55	--	
Late adolescence	0.50	0.64	0.27	--
Young Adulthood	0.42	0.82	0.99	0.67

* Significance reached with Bonferroni-corrected alpha. *Preschool* includes participants < 7 years old, *childhood* includes those ≥ 7 to < 13 years old, *adolescence* includes those ages ≥ 13 to < 18 years old , *late adolescence* includes those ages ≥ 18 to < 23 years old, and *young adulthood* includes those ≥ 23 to 30 years old.

© 2024 Copeland WE et al. *JAMA Network Open.*

**eTable 5.** Comparison of Event-Symptoms Associations Between Developmental Period Groups For Females (Includes All Samples)				
	Preschool	Childhood	Adolescence	Late adolescence
**Stressful Events**	*p*	*p*	*p*	*p*
Preschool	--			
Childhood	0.78	--		
Adolescence	0.62	0.86	--	
Late adolescence	0.15	0.23	0.14	--
Young Adulthood	0.002*	0.004	0.003	0.10
**Traumatic Events**				
Preschool	--			
Childhood	0.77	--		
Adolescence	0.84	0.49	--	
Late adolescence	0.40	0.46	0.14	--
Young Adulthood	0.76	0.92	0.63	0.67

* Significance reached with Bonferroni-corrected alpha. *Preschool* includes participants < 7 years old, *childhood* includes those ≥ 7 to < 13 years old, *adolescence* includes those ages ≥ 13 to < 18 years old , *late adolescence* includes those ages ≥ 18 to < 23 years old, and *young adulthood* includes those ≥ 23 to 30 years old.

© 2024 Copeland WE et al. *JAMA Network Open.*

**eTable 6.** Comparison of Event-Symptoms Associations Between Developmental Groups for Males				
	Preschool	Childhood	Adolescence	Late adolescence
**Stressful Events**	*p*	*p*	*p*	*p*
Preschool	--			
Childhood	0.63	--		
Adolescence	0.71	0.88	--	
Late adolescence	0.71	0.99	0.91	--
Young Adulthood	0.07	0.10	0.10	0.15
**Traumatic Events**				
Preschool	--			
Childhood	0.58	--		
Adolescence	0.39	0.69	--	
Late adolescence	0.16	0.18	0.36	--
Young Adulthood	0.90	0.69	0.46	0.20

* Significance reached with Bonferroni-corrected alpha. *Preschool* includes participants < 7 years old, *childhood* includes those ≥ 7 to < 13 years old, *adolescence* includes those ages ≥ 13 to < 18 years old , *late adolescence* includes those ages ≥ 18 to < 23 years old, and *young adulthood* includes those ≥ 23 to 30 years old.

© 2024 Copeland WE et al. *JAMA Network Open.*

**eTable 7.** Follow-Up Model Testing Additive and Interactive Effects of Traumatic and Stressful Events						
	Traumatic events		Stressful events		Interaction	
	B	*SE*	*B*	*SE*	*B*	*SE*
Preschool	--	--	--	--	--	--
Childhood	0.22*	0.05*	0.15	0.06	0.02	0.03
Adolescence	0.29*	0.05*	0.23*	0.06*	-0.05	0.14
Late adolescence	0.24*	0.07*	0.30*	0.09*	-0.04	0.04
Young Adulthood	0.36*	0.08*	0.78*	0.16*	-0.22	0.12

* Significance reached with Bonferroni-corrected alpha. *Preschool* includes participants < 7 years old, *childhood* includes those ≥ 7 to < 13 years old, *adolescence* includes those ages ≥ 13 to < 18 years old , *late adolescence* includes those ages ≥ 18 to < 23 years old, and *young adulthood* includes those ≥ 23 to 30 years old.

© 2024 Copeland WE et al. *JAMA Network Open.*
