# Supplementary material for: Negative Life Events and Emotional Symptoms From Ages 2 to 30 Years
Source: JAMA Netw Open. 2024 Aug 29;7(8):e2429448. doi: 10.1001/jamanetworkopen.2024.29448 (PMC11362870; doi:10.1001/jamanetworkopen.2024.29448)
Supplement: Supplement 2. — Data Sharing Statement [file jamanetwopen-e2429448-s002.pdf]

Data Sharing Statement

Copeland. Negative Life Events and Emotional Symptoms From Ages 2 to 30 Years. *JAMA Netw Open*. Published August 29, 2024. doi:10.1001/jamanetworkopen.2024.29448

Data
Data available: Yes
Data types: Deidentified participant data
How to access data: For data-sharing requests, please contact Bill Copeland (william.copeland@med.uvm.edu)
When available: With publication

Supporting Documents
Document types: None

Additional Information
Who can access the data: Researchers whose proposed use of the data has been approved
Types of analyses: For any purpose
Mechanisms of data availability: After approval of a proposal
